# Supplementary material for: Molecular characterization of clonal lineage and staphylococcal toxin genes from S. aureus in Southern Nigeria
Source: PeerJ. 2018 Jul 9;6:e5204. doi: 10.7717/peerj.5204 (PMC6042479; doi:10.7717/peerj.5204)
Supplement: Data S1 [file peerj-06-5204-s001.docx]

Figure 1 Occurence of spa type

t008 1 2%

t021 1 2%

t050 1 2%

t069 5 11%

t084 1 2%

t091 8 17%

t095 1 2%

t127 5 11%

t1045 3 6%

t1095 2` 4%

t1154 1 2%

t1171 1 2%

t1931 1 2%

t292 1 2%

t311 1 2%

t318 1 2%

t355 8 17%

t537 1 2%

t786 1 2%

t939 2 4%

t14223 1 2%

Figure 2: Percentage frequency of staphylococci enterotoxin genes

Pvl 23%

SeA 21%

SeO 34

SeM 17

SeQ 13

SeN 9

SeK 13

SeP 19

SeL 4

SeB 13

SeG 30

SeR 2

SeU 4

SeI 11

SeH 6

Figure 3: Association of staphylococci enterotoxin genes with spa types

| No |  | Toxins | | | | | | | | | | | | | | |  |
| --- | --- | --- | --- | --- | --- | --- | --- | --- | --- | --- | --- | --- | --- | --- | --- | --- | --- |
| N0 | spa | pvl | A | O | M | Q | N | K | P | L | B | G | R | U | I | H |  |
| S. aureus FA001 | t355 | + |  |  |  |  |  |  |  |  |  |  |  |  |  |  | 1 |
| S. aureus FA002 | t537 |  |  | + | + |  |  |  |  | + |  | + |  |  |  |  | 4 |
| S. aureus FA003 | t355 |  |  |  |  |  |  |  |  |  |  |  |  |  |  |  | 0 |
| S. aureus FA004 | t355 | + |  |  |  |  |  |  |  |  |  |  |  |  |  |  | 1 |
| S. aureus FA005 | t355 | + |  |  |  |  |  |  |  |  |  |  |  |  |  |  | 1 |
| S. aureus FA006 | t1931 | + |  |  |  |  |  |  |  |  |  |  |  |  |  |  | 1 |
| S. aureus FA007 | t355 | + |  |  |  |  |  |  |  |  |  |  |  |  |  |  | 1 |
| S. aureus FA008 | t355 | + |  |  |  |  |  |  |  |  |  |  |  |  |  |  | 1 |
| S. aureus FA009 | t355 | + |  |  |  |  |  |  |  |  |  |  |  |  |  |  | 1 |
| S. aureus FA010 | t355 | + |  |  |  |  |  |  |  |  |  |  |  |  |  |  | 1 |
| S. aureus FA012 | t1045 |  |  | + | + |  |  |  |  |  |  | + |  |  |  |  | 3 |
| S. aureus FA013 | t021 | + |  | + |  |  |  |  |  |  |  | + |  | + |  |  | 4 |
| S. aureus FA014 | t069 |  | + |  |  | + |  | + |  |  | + |  |  |  |  |  | 4 |
| S. aureus FA015 | t1095 |  |  | + | + |  |  |  |  |  |  | + |  |  |  |  | 3 |
| S. aureus FA016 | t1095 |  |  | + | + |  |  |  |  |  |  |  |  |  |  |  | 2 |
| S. aureus FA017 | t069 |  | + |  |  | + |  | + |  |  | + |  |  |  |  |  | 4 |
| S. aureus FA018 | t069 |  | + |  |  | + |  | + |  |  | + |  |  |  |  |  | 4 |
| S. aureus FA019 | t14223 |  |  |  |  |  |  |  |  |  |  |  |  |  |  |  | 0 |
| S. aureus FA020 | t095 |  |  | + | + |  |  |  |  | + |  | + |  |  |  |  | 4 |
| S. aureus FA021 | t091 |  |  |  |  |  |  |  | + |  |  |  |  |  |  |  | 1 |
| S. aureus FA022 | t069 |  | + |  |  | + |  | + |  |  | + |  |  |  |  |  | 4 |
| S. aureus FA023 | t091 |  |  |  |  |  |  |  | + |  |  |  |  |  |  |  | 1 |
| S. aureus FA024 | t292 |  |  | + | + |  |  |  |  |  | + | + | + |  |  |  | 5 |
| S. aureus FA025 | t939 |  |  | + | + |  |  |  |  |  |  | + |  |  |  |  | 3 |
| S. aureus FA026 | t318 | + |  | + |  |  |  |  |  |  |  | + |  | + |  |  | 4 |
| S. aureus FA027 | t069 |  | + |  |  | + |  | + |  |  | + |  |  |  |  |  | 4 |
| S. aureus FA028 | t050 |  |  | + | + |  |  |  |  |  |  | + |  |  |  |  | 3 |
| S. aureus FA029 | t1171 |  |  |  |  |  |  |  |  |  |  |  |  |  |  |  | 0 |
| S. aureus FA031 | t091 |  |  |  |  |  |  |  | + |  |  |  |  |  |  |  | 1 |
| S. aureus FA034 | t084 |  |  |  |  |  |  |  |  |  |  |  |  |  |  |  | 0 |
| S. aureus FA035 | t091 |  |  | + |  |  |  |  | + |  |  |  |  |  |  |  | 2 |
| S. aureus FA036 | t1045 |  |  | + |  |  |  |  |  |  |  | + |  |  | + |  | 3 |
| S. aureus FA037 | t1045 |  |  | + |  |  | + |  |  |  |  | + |  |  | + |  | 4 |
| S. aureus FA039 | t127 | + | + |  |  | + |  | + |  |  |  |  |  |  |  | + | 5 |
| S. aureus FA040 | t939 |  |  | + |  |  | + |  |  |  |  | + |  |  | + |  | 4 |
| S. aureus FA041 | t311 |  |  | + |  |  | + |  |  |  |  | + |  |  | + |  | 4 |
| S. aureus FA043 | t127 |  | + |  |  |  |  |  |  |  |  |  |  |  |  | + | 2 |
| S. aureus FA044 | t786 |  |  |  |  |  |  |  |  |  |  |  |  |  |  |  | 0 |
| S. aureus FA045 | t091 |  |  |  |  |  |  |  | + |  |  |  |  |  |  |  | 1 |
| S. aureus FA046 | t091 |  |  |  |  |  |  |  | + |  |  |  |  |  |  |  | 1 |
| S. aureus FA047 | t127 |  | + |  |  |  |  |  |  |  |  |  |  |  |  | + | 2 |
| S. aureus FA048 | t091 |  |  |  |  |  |  |  | + |  |  |  |  |  |  |  | 1 |
| S. aureus FA049 | t127 |  | + |  |  |  |  |  |  |  |  |  |  |  |  |  | 1 |
| S. aureus FA050 | t1154 |  |  | + |  |  | + |  |  |  |  | + |  |  | + |  | 4 |
| S. aureus FA051 | t127 |  | + |  |  |  |  |  |  |  |  |  |  |  |  |  | 1 |
| S. aureus FA052 | t008 |  |  |  |  |  |  |  | + |  |  |  |  |  |  |  | 1 |
| S. aureus FA053 | t091 |  |  |  |  |  |  |  | + |  |  |  |  |  |  |  | 1 |
| no |  | 11 | 10 | 16 | 8 | 6 | 4 | 6 | 9 | 2 | 6 | 14 | 1 | 2 | 5 | 3 |  |
| % |  | 23 | 21 | 34 | 17 | 13 | 9 | 13 | 19 | 4 | 13 | 30 | 2 | 4 | 11 | 6 |  |
